# Study of nitrate levels in fruits and vegetables to assess the potential health risks in Bangladesh

**DOI:** 10.1038/s41598-021-84032-z

**Published:** 2021-02-25

**Authors:** Rayhan Uddin, Mostak Uddin Thakur, Mohammad Zia Uddin, G. M. Rabiul Islam

**Affiliations:** 1 Department of Food Engineering and Tea Technology, Shahjalal University of Science and Technology, Sylhet , 3114 Bangladesh; 2 Department of Analytical Chemistry and Environmental Science, Training Institute for Chemical Industries, Narsingdi, 1611 Bangladesh; 3 Delta Pharma Limited, Pakundia Plant, Kishoreganj, 2300 Bangladesh

**Keywords:** Chemical biology, Health care

## Abstract

Nitrate is a chemical compound naturally present in fruits and vegetables. This study aims at assessing the nitrate levels and health risks arising from high consumption of fruits and vegetables in Bangladesh. Sixteen species of fruits and vegetables were examined for nitrates using High-Performance Liquid Chromatography with Photo Diode Array (PDA) detector. Ward’s hierarchical cluster analysis was carried out to identify the cluster of tested fruits and vegetables for the nitrate contents. A point estimate of the daily intake was applied to find the health risks that arise due to elevated levels of nitrate in fruits and vegetables. The results show that root and tuber vegetables accumulate significantly higher levels of nitrate in comparison to fruits and fruit vegetables (P < 0.05**).** In cluster analysis, the nitrate accumulation of fruits and vegetables show four clear clusters contributing to 29.54%, 7.17%, 4.42%, and 58.57% of the total nitrate content in the entire sample. The risk assessment of the Estimated Daily Intake (EDI) and Health Risk Index (HRI) of almost all the tested samples was in the acceptable range, except for radish, thereby indicating the acceptance of risk due to nitrate intake in Bangladesh. As nitrate may have had risk factor for health, during cultivation and storing the product should be properly monitored.

## Introduction

Dietary inorganic nitrates have had a notoriously bad reputation for more than 50 years^1^. Currently, the concentration of nitrates in fruits and vegetables are exceeding the allowed limits worldwide^2,3^. This has led to strict regulations regarding the allowable levels of nitrate in our food based on the assumption that nitrate is overall harmful to our health. Concerns over nitrate intake originate from the fact that they are associated with the genesis of some forms of cancer and methemoglobinemia throughout the world^4,5^. They react with the secondary amines in the stomach and lead to the formation of nitrosamines (N-nitroso compounds), some of which are known to be carcinogenic, teratogenic, and mutagenic. This increases the risk of cancer to the stomach and esophagus^6–9^. Some studies have evidence that a positive correlation exists between high nitrate consumption and gastric cancer in humans^10,11^. On the other hand, several plausible health benefits and preventive effects of nitrate have also been reported recently. A substantial number of studies in health and hypertensive subjects have now shown that nitrate has beneficial cardiovascular effects, including lowering of blood pressure^12–17^. Scientific evidence also suggests that having abundant vitamin C and polyphenol in fruits and vegetables facilitate in the nonenzymatic reduction of toxic nitrite to beneficial nitric oxide, thereby reducing the chances of nitrite reacting with secondary amines forming nitrosamines^18,19^. Therefore, it has been pointed out that the evidence for adverse effects of nitrate is inconsistent, and nitrate may actually be beneficial. Hence, the concepts of risk–benefit of exposure to dietary nitrate become a challenging/unresolved issue to the scientists.

Furthermore, Fruits and vegetables play an important role in alleviating micronutrient deficiencies and associated health repercussions, for example, lowering the risk of cancer, cardiovascular disease, and mortality^20–24^. Therefore, the Food and Agriculture Organization (FAO) and World Health Organization (WHO) recommend consuming at least 400 g/day of fruits and vegetables^25^. On the one hand, the per-capita consumption of fruits and vegetables in Bangladesh is substantially lower than the WHO norms, and there is an urgent need to encourage local value chains to deliver safe and affordable produce to the wider population^26^. On the other hand, nitrate pollution from chemical fertilizers, pesticide residue (PR) overload in vegetables, and microbiological contamination along the value chain have emerged as alarming public health issues^27^. Some studies have shown that leafy vegetables and fruits contain higher levels of nitrate and contribute to about 85% of the dietary intake of nitrates in a number of societies^20–22^.The application of excessive nitrogen fertilizers to grow fruits and vegetables is one of the key factors for the accumulation of nitrates in fruits and vegetables^28^. Besides, the presence of nitrates in the produce also depends on a number of factors that can vary greatly from region to region such as biological properties of crop, lighting conditions, soil properties, humidity, frequency of planting in the field, vegetation period, season of harvest, processing time, geographical region and fertilization^29–32^.

The concept of hazard characterisation of micronutrients must consider that adverse effects may arise from intakes that are too low (deficiency) as well as too high (toxicity)^33^. The highest daily dose, at which no adverse effects are observed in the most susceptible animal species or model system during chronic exposure, is identified as the No Observable Adverse Effect Level (NOAEL). The NOAEL value is used as the basis for setting human safety standards for chemicals present in our diet, such as the TDI or the ADI. Being an agriculture-based country, Bangladesh grows a variety of fruits and vegetables throughout the year. However, it is true that in Bangladesh, there is a lack of information available for the levels of nitrate concentration in fruits and vegetables. Therefore, in this study, we aim to determine the concentration of nitrates in fruits and vegetables available in the local market as well as assess the health risk to humans upon consumption of the nitrate contaminated fruits and vegetables based on the Acceptable Daily Intake (ADI) and Health Risk Index (HRI), along with the established safety limits.

## Materials and methods

### Study area and sample collection

**Figure 1:**
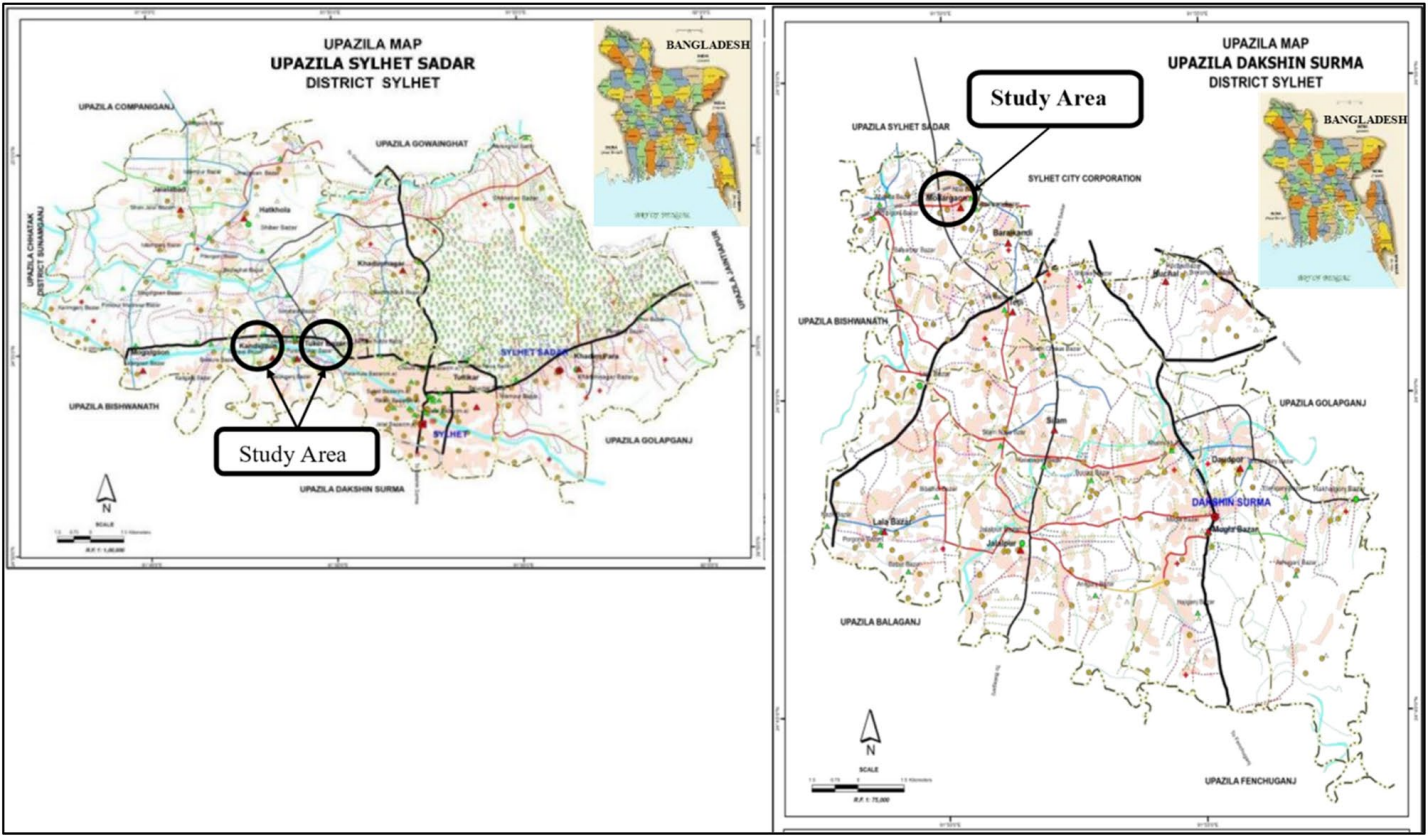
The study areas: Tuker Bazar and Kandergaon in Sylhet Sadar Upazila (left panel) and Mollargaon in Dakshin Surma Upazila (right panel) Source :(http://www.lged.gov.bd/ViewMap.aspx).

The preliminary phase of this study was conducted at Tuker Bazar, Kandigaon in Sylhet Sadar Upazila (left panel) and Mollargaon in Dakshin Surma Upazila (right panel) areas, which are the three major agriculturally productive regions in Sylhet division in Bangladesh (see Fig. 1). The longitudes selected were 24.9341° N and 91.8660° E for Tuker Bazar, 24.9111° N and 91.8075° E for Kandigaon, and 24.8753° N and 91.8270° E for Mollargaon.

The sites selected were based on crops grown, fertilizers used as well as opinions from local people and agricultural officers. The vegetables and fruits were selected based on two assumptions: (i) High consumption rate of fruits and vegetables among Bangladeshi people, and (ii) high nitrate content in fruits and vegetables grown in Bangladesh. All the samples were selected randomly at the time of their biological maturity during the growing season (May 2019 to December 2019). The samples were grouped into three viz., (i) root and tuber vegetables, (ii) fruit vegetables, and (iii) fruits. The samples were placed in a plastic bag and transferred to the laboratory after being labeled. The samples were kept overnight at a temperature below 4 °C until the analysis on the following day. The protocol of the study was approved by the Institutional Review Board of Shahjalal University of Science and Technology, Sylhet, Bangladesh No. AS/2019/2/33).

### Reagents and standards

Analytical grade potassium nitrate (Merck, Germany), hydrochloric acid (Sigma Aldrich), HPLC grade methanol and 1-Pentanesulfonic acid sodium salt were purchased from an online supplier. Deionized water was used for preparing all the solutions and also for sample extraction.

### Sample preparation and extraction

The sample preparation and extraction were conducted according to the method developed by Hongsibsong et al.^34^. The non-edible parts were removed, and the samples were subjected to cutting and homogenization using a cutter and homogenizer, respectively. Then, the homogenized samples were immediately stored at –20 °C before the analysis. 50 mL of deionized water was added to 2 g of the homogenized sample in a 100 mL volumetric flask. The flask was then moved to a boiling water bath for 20 min at 80 °C, shaken up and laid on the table to cool down, and further diluted to a final volume of 100 mL with deionized water. The first filtrate of 3 mL was discarded, and the rest was stored for the determination of nitrate. All the samples were analyzed within 1 h of preparation and extraction.

### Determination of nitrate

The nitrate in the present study was analyzed according to the method described by Hsu et al.^35^ with some modifications. High-Performance Liquid Chromatography (HPLC) (Shimadzu HPLC Prominence-***i*** LC-2030 LT) with Photo Diode Array (PDA) detector was used to detect the nitrate. The separation was performed on a C_18_ column (ZORBAX Eclipse XDB-C_18_, 80 Å, 250 × 4.6 mm, 5 µm (Agilent Technologies).

The buffer solution used for the analytical procedures was prepared by dissolving 1.74 g of 1-Pentanesulfonic acid sodium salt in 950 mL deionized water and then added into a 1000 mL beaker; deionized water was used to dilute the solution up to the mark. KNO_3_ (162.79 mg) was weighed in a 100 mL volumetric flask and then dissolved in deionized water. Subsequent serial dilutions were made to achieve standards used for preparing the calibration curve of nitrate. The calibration curve was obtained by injecting five different concentrations of standard nitrate solutions of 100, 200, 300, 400, and 500 ppm and plotting peak area against the concentration (ppm). The curve showed a high degree of linearity, where the slope appears at 13,690.2 with an average correlation coefficient, R^2^ = 0.9962. The Relative Standard Deviation (RSD) for peak areas was below 1%. Therefore, we used the calibration curve to calculate the concentration of nitrate in selected fruits and vegetable samples and finally reported the values as mg kg^–1^.

The mobile phase solution consisting of methanol (30%) and buffer (70%) was allowed to pass through the HPLC column until a stable baseline signal was equilibrated. The pH of the mobile phase was adjusted to 2.8 with 2 M hydrochloric acid solution. The injection volume was 10 µL with the flow rate set at 1 mL/min and wavelength set at 225 nm. The column oven temperature was 40 ^0^C, and the run time was 10 min. As soon as the injections of the standard solution gave reproducible retention times and peak areas, each sample solution was injected for analysis. The peaks of the sample were identified by comparison with the respectable peaks of the standards.

### Health risk estimation

The nitrate that enters into the body through fruits and vegetables becomes a substance of concern if it exceeds its Acceptable Daily Intake (ADI) limit or toxicity level, which may then lead to death^36^. The ADI for dietary nitrates is 3.7 mg/kg body weight according to the regulations of Joint Expert Committee of Food and Agriculture (JECFA) and the European Commission's Scientific Committee on Food (SCF)^37–39^.

The daily intake of nitrate was calculated to estimate the average daily nitrate accumulation in a person of specific bodyweight and also to estimate the relative bioavailability of nitrate. The Estimated Daily Intake (EDI) does not take into account the possible metabolic excretion of the nitrate and considers only the possible ingestion rate. The EDI was calculated based on the following equation

1 $$\begin{aligned} & {\text{EDI}} \\ & \quad = \frac{{{\text{Average }}\,{\text{daily}}\,{\text{ consumption}}\,{\text{ of }}\,{\text{fruits }}\,{\text{and }}\,{\text{vegetables }} \times {\text{ concentration}}\,{\text{ of }}\,{\text{nitrate }}\,{\text{in }}\,{\text{fruits }}\,{\text{and}}\,{\text{ vegetables}}\,{ }\left( {{\text{mg}}/{\text{kg}}} \right)}}{{{\text{Body }}\,{\text{weight}}}} \\ \end{aligned}$$

In Eq. (1) the average (per capita) consumption of vegetables and fruits was considered as 232 and 35.5 g, respectively, as per the recent *Household Income and Expenditure Survey*^26^. We also calculated the EDI and HRI if a person consumed 400 g of fruits and vegetables as per the WHO and FAO guidelines^40^. In both cases, the body weight of 60 kg was taken for adults and 27 kg for children^41^. The calculated EDI was then compared with the ADI for the assessment of health hazards associated with the consumption of nitrate-containing fruits and vegetables.

### Health Risk Index (HRI)

An HRI > 1 for any nitrate in food products means that the consumer population is at a potential health risk. The value of the Health Risk Index (HRI) depends on the EDI value of the food products and the oral Reference Dose (RfD). The oral RfD is the numerical estimate of the daily oral exposure of nitrate to humans that is not likely to cause harmful effects during the lifetime, including sensitive subgroups such as children^42^. The HRI for nitrate exposure due to consumption was calculated using the Eq. (2) reported by previous investigations^43,44^

2 $${\text{HRI}} = \frac{EDI}{{Rfd}}$$

where EDI is for Estimated Daily Intake (mg/kg body weight/day), and Rfd is the reference dose (mg/kg body weight/day). The oral Rfd of the nitrate nitrogen is 1.6 mg/kg body weight/day, which is equivalent to 7.09 mg/kg body weight/day of nitrate according to the United States Environmental Protection Agency (USEPA) USEPA^45^.

### Statistical analysis

All experiments were carried out in three replicates. The Shapiro–Wilk test is applied to determine the normal distribution of nitrate and EDI values**.** Since the data were not normally distributed, the Kruskal–Wallis test was performed to analyze the relative nitrate content of different food groups. The Mann–Whitney U test with Bonferroni correction was applied for the comparison of each group. The analysis was performed using R software for windows version (version 4.0.2). The *P* values of < 0.05 were considered to indicate statistical significance. Ward’s hierarchical cluster analysis was used to categorize the tested fruits and vegetables in terms of their nitrate content.

## Results

### Nitrate in fruits and vegetables

Fruits and vegetables are recognized to provide a significant portion of nitrates in the nutritional regime. The samples analyzed in this present study were categorized and presented with their respective nitrate content in the previously mentioned groups of three.

**Figure 2:**
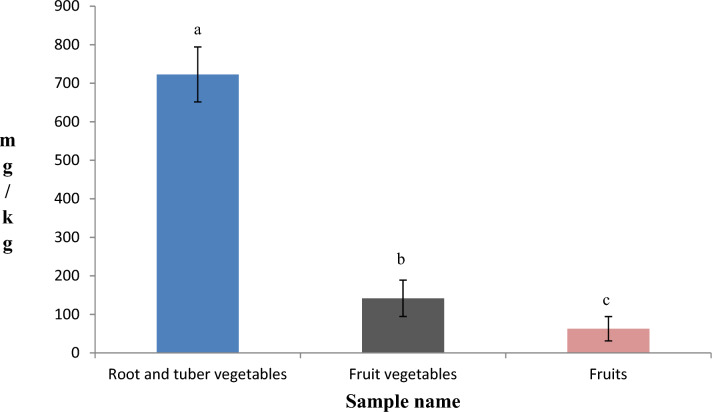
Nitrate levels in different tested root and tuber vegetables, fruit vegetables, and fruit samples. Error bars represent standard deviation. Columns that have the same letter are not significantly different at P-value = 0.05.

Nitrates were detected in all the tested fruits and vegetables, and the results show that there is a significant difference in nitrate concentration among the root and tuber vegetables, fruit vegetables, and fruits (p < 0.05). The nitrate content in root and tuber vegetables (722.80 mg/kg) was found to be significantly higher than that of fruit vegetables (141.75 mg/kg) (Fig. 2). In addition, lower nitrate content was observed in the fruit samples (62.74 mg/kg) when compared to the vegetables.

As presented in Table 1, among the root and tuber vegetables, the highest mean concentration of nitrate was determined in radish (2501.24 mg/kg) followed by potato (307.19 mg/kg) and carrot (36.80 mg/kg). In the case of fruit vegetables, brinjal scored the highest nitrate content followed by cauliflower and cucumber (> 200 mg/kg). The nitrate concentration in lady finger and multitude appears within a magnitude of 100 ~ 150 mg/kg. The nitrate concentration among other fruit vegetables appears below 100 mg/kg. In the case of fruits, it was observed that the mean nitrate concentration of banana scored the highest (200 mg/kg), whereas other three fruits viz., apple, grape, and orange scored below 20 mg/kg.

**Table 1:** Mean concentrations of nitrate in the tested fruits and vegetables.

Fruits and vegetables	Nitrate ± S.D. (mg/kg)
**Root and tuber vegetables**	
Carrot	36.80 ± 3.06
Potato	307.19 ± 47.23
Radish	2501.24 ± 342.81
**Fruit vegetables**	
Tomato	43.19 ± 31.15
Cucumber	214.57 ± 14.45
Cauliflower	259.58 ± 7.81
Brinjal	284.26 ± 7.98
Bean	71.08 ± 5.09
Bitter Guard	46.52 ± 3.38
Lady finger	124.48 ± 6.26
Pumpkin	20.23 ± 2.45
Multitude	110.43 ± 9.73
**Fruits**	
Banana	196.09 ± 138.82
Apple	17.92 ± 2.52
Grape	19.07 ± 3.07
Orange	17.90 ± 1.95

**Figure 3:**
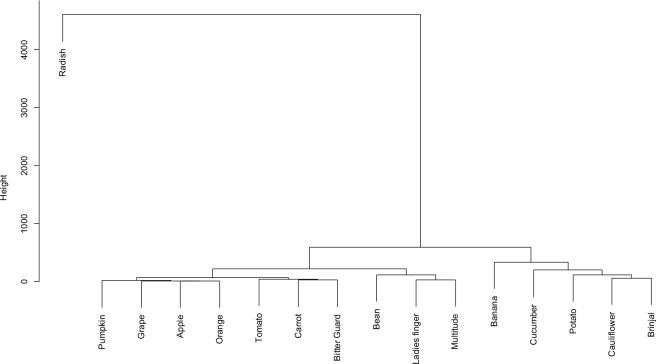
Dendrogram showing hierarchical clustering for nitrate content.

The cluster results of nitrate accumulation in fruits and vegetables are presented in Fig. 3. As seen from this figure, the tested fruits and vegetables are categorized into four clear clusters. The first cluster includes brinjal, cauliflower, potato, cucumber, and banana. The second cluster includes fruit vegetables like lady finger and beans. The pumpkin, grape, apple, orange, tomato, carrot, and bitter guard comprise the third cluster, whereas the fourth cluster includes only radish. The first, second, third, and fourth groups are responsible for 29.54%, 7.17%, 4.42%, and 58.57% of the total nitrate content, respectively.

### Health risk assessment

There is a direct correlation between the degree of toxicity of nitrate and daily intake. The health risk exposure of nitrate was calculated as Estimated Daily Intake (EDI) and Health Risk Index (HRI) in this study according to Eq. (1) and (2), and the outcomes were presented in Table 2.

**Table 2:** Estimated Daily Intake (EDI) and Health Risk Index (HRI) for the Fruits and vegetables in accordance with the current daily consumption and WHO/FAO recommendation for adult and children.

Fruit and vegetable samples	According to current daily consumption						According to WHO recommendation of consumption of 400 g/day					
	EDI (mg NO_3/_Kg Body weight/day)		%ADI		HRI		EDI (mg NO_3/_Kg Body weight/day)		%ADI		HRI	
	Adult	Children	Adult	Children	Adult	Children	Adult	Children	Adult	Children	Adult	Children
**Root and tuber vegetables**												
Carrot	0.14	0.31	3.78	8.38	0.02	0.04	0.25	0.55	6.76	14.86	0.04	0.08
Potato	1.19	2.64	32.16	71.35	0.17	0.37	2.04	**4.55**	55.14	–	0.29	0.64
Radish	**9.61**	**21.49**	–	–	**1.36**	**3.03**	**16.67**	**37.06**	–	–	**2.35**	**5.23**
**Fruit vegetables**												
Tomato	0.17	0.37	4.59	10	0.02	0.05	0.29	0.64	7.84	17.29	0.04	0.09
Cucumber	0.83	1.84	22.43	49.73	0.12	0.26	1.43	3.18	38.65	85.94	0.20	0.45
Cauliflower	1.00	2.23	27.03	60.27	0.14	0.31	1.73	**3.85**	46.76	–	0.24	0.54
Brinjal	1.10	2.44	29.73	65.95	0.16	0.34	1.90	**4.21**	51.35	–	0.27	0.59
Bean	0.27	0.61	7.30	16.49	0.04	0.09	0.47	1.05	12.70	28.38	0.07	0.15
Bitter Guard	0.18	0.40	4.86	10.81	0.03	0.06	0.31	0.69	8.38	18.65	0.04	0.10
Lady finger	0.48	1.07	12.97	28.92	0.07	0.15	0.83	1.84	22.43	49.73	0.12	0.26
Pumpkin	0.08	0.17	2.16	4.59	0.01	0.02	0.13	0.30	3.51	8.11	0.02	0.04
Multitude	0.43	0.95	11.62	25.68	0.06	0.13	0.74	1.64	20	44.32	0.10	0.23
**Fruits**												
Banana	0.12	0.26	3.24	7.03	0.02	0.04	1.30	2.91	35.14	78.64	0.18	0.41
Apple	0.01	0.02	0.27	0.54	0.001	0.002	0.12	0.27	3.24	7.30	0.02	0.04
Grape	0.01	0.03	0.27	0.81	0.001	0.004	0.13	0.28	3.51	7.57	0.02	0.04
Orange	0.01	0.02	0.27	0.54	0.001	0.002	0.12	0.27	3.24	7.30	0.02	0.04

Among the tested root and tuber vegetables, the highest EDI of nitrate appears in radish for adults followed by potato and carrot in both situations, i.e., as per current consumption and WHO recommendation. The Health Risk Index exceeds the accepted limit of 1 for adults and children only in the case of radish. The estimated daily intake of nitrate due to consumption of fruit vegetables like cucumber and cauliflower appears to be around 1 mg NO_3_/Kg body weight for adults and 2 mg NO_3_/Kg body weight for children. The value varies between 3 – 4 mg NO_3_/Kg body weight for children if the consumption is as per the WHO recommendation. For the other fruit vegetables, the current estimated daily intake is below 1 mg NO_3_/Kg body weight for adults. The EDI varies between 0.10—3 mg NO_3_/Kg body weight in the case of bananas for adults and children. For all the tested fruits and vegetables, the HRI is within its acceptable limit, i.e., less than 1.

## Discussion

The results show that the highest content of nitrate was found in the root and tuber vegetables, followed by fruit vegetables and fruits. The reason for fruits containing lower concentrations of nitrate might be the use of less fertilizers in fruit cultivation in Bangladesh. These results coincide with the findings of Bahadoran et al., study from Iran ^22^; Susin, kmecl and Gregorcic’s study from Slovenia^46^.

From this study, it is clear that the mean concentration values of nitrate (mg/kg) detected in fruits and vegetables of Bangladesh were lower than those reported in other studies^22,47–52^. The nitrate content of the brinjal, beans, ladies finger and bitter guard were similar to the findings of Taghipour et al.^53^ and Taneja et al.^54^. Bangladesh has a tropical monsoon climate, which is characterized by wide seasonal variations in rainfall, high temperatures along with long sunshine periods. As reported by Escobar-Gutierrez et al.^55^, lower levels of nitrate in fruits and vegetables were related to high temperatures and longer sunshine periods. This may explain the overall lower levels of nitrate obtained in the present study. In contrast, a high concentration of nitrates and high variability between the findings, which contribute to the high standard deviation in some vegetable and fruit samples may be due to the soil type, use of fertilizers, time of harvesting, groundwater nitrate pollutions and agricultural practices^56–58^. However, further investigation is required in this regard.

It appears that the EDI values for potato, tomato, carrot, cucumber, cauliflower from our studies were higher in comparison to the results of Gruszecka-Kosowska and Baran (0.84, 0.04, 0.03, 0.16, 0.03 mg NO_3_/Kg body weight, respectively) from Poland^59^. Mehri et al.^60^ in a study from Iran, reported high EDI values for carrot (0.19 mg NO_3_/Kg body weight) and tomato (0.17 mg NO_3_/Kg body weight ), which are similar to our findings. However, the EDI values reported for the potato samples (0.13 mg NO_3_/Kg body weight) were lower than the results obtained in our study ^60^.

Using the standard value of 3.7 mg/kg body weight/day as the benchmark of ADI threshold (as per the guidelines of WHO)^37^; and HRI < 1 (as the benchmark of HRI threshold), it was found that radish exceeds the permissible limit for both adults and children, indicating risks from nitrates. Several previous studies viz. Gruszecka-Kosowska and Baran from Poland, Suh et al. from Korea and Sebaeia and Refai from Egypt have demonstrated that no tested entity exceeds the standard value of ADI^59,61,62^. The reason for radish exceeding the threshold value of ADI in our study might be due to the intensity of fertilization, high nitrate accumulation rates, or the use of nitrate contaminated water.

The estimated daily intake and health risk index for each fruit and vegetable was calculated based on a daily consumption rate of 400 g. In connection to this, the maximum permissible limit of 3.7 mg/kg body weight/day ADI as per the guidelines of WHO was found to exceed in radish for adults and radish, potato, cauliflower, and brinjal for children; Whereas for HRI only the radish was found to exceed the permissible limit.

Hence, this study indicates the adverse effects of nitrate pollution in fruits and vegetables in Bangladesh is acceptable. However, the reason for getting comparatively lower EDI and HRI values might be due to lesser contamination of fruits and vegetables with nitrate as well as lower than the recommended intake of fruits and vegetables (400 g/person/day)^63^.

It should be kept in mind that consumption of fruits and vegetables is not the only route (though prime source) for nitrate to enter our body, and a Health Risk Index of less than 1 cannot alone indicate a safe and healthy level of nitrate intake. Therefore, other probable sources of nitrate, such as drinking water and other foodstuffs should be studied to determine the health risks of nitrate.

### Limitations of the study

We have studied nitrate level in a few popular F&V (fruits and vegetables) samples, which were available during our study time frame. This small-scale analysis does not represent the complete scenario of nitrate content in F&V in Bangladesh. Therefore, more investigations need to be conducted on a large scale with a larger sample size to get the overall idea about the levels of nitrate available in fruits and vegetables in Bangladesh.

## Conclusion

The prime concern for nitrate-rich diet is the endogenous formation of carcinogenic nitrosamines. Vegetables and fruits are considered the first contributors to dietary nitrate. This study clearly demonstrates that the average nitrate concentration in almost all samples was comparatively lower than that of the standard level. However, we must keep in mind that prolonged and inefficient storage, along with excessive use of chemical fertilizers and nitrate polluted water, contributes to elevated levels of nitrate in fruits and vegetables. Excluding radish, the HRI values of nitrate for other samples were < 1, which indicates that health risks associated with nitrate exposure were not significant.

Therefore, the intake of nitrate through fruits and vegetables could be considered safe for the consumers. This is the very first study carried out to quantify the nitrate levels and assess the associated health risks in Bangladesh which will provide useful data to industry and health professionals.
