# Author Correction: Study of nitrate levels in fruits and vegetables to assess the potential health risks in Bangladesh

**DOI:** 10.1038/s41598-025-02766-6

**Published:** 2025-06-13

**Authors:** Rayhan Uddin, Mostak Uddin Thakur, Mohammad Zia Uddin, G. M. Rabiul Islam

**Affiliations:** 1 Department of Food Engineering and Tea Technology, Shahjalal University of Science and Technology, Sylhet , 3114 Bangladesh; 2 Department of Analytical Chemistry and Environmental Science, Training Institute for Chemical Industries, Narsingdi, 1611 Bangladesh; 3 Delta Pharma Limited, Pakundia Plant, Kishoreganj, 2300 Bangladesh

Correction to: *Scientific Reports* 10.1038/s41598-021-84032-z, published online 25 February 2021

The original version of this Article contained an error in Reference 4, which was incorrectly given as:

Vickers, N. J. Animal communication: When i’m calling you, will you answer too?. Curr. Biol. 27(14), R713–R715 (2017).

The correct reference is listed below

Vermeer IT, Van Maanen JM. Nitrate exposure and endogenous formation of carcinogenic nitrosamines in humans. Rev. Environ. Health. 16(2), 105-16 (2001)

The original Article has been corrected.

